# Effect of Rooibos (*Aspalathus linearis*) extract on atorvastatin‐induced toxicity in C3A liver cells

**DOI:** 10.1002/jcp.29756

**Published:** 2020-05-27

**Authors:** Danielle A. Millar, Sandra Bowles, Shantal Lynn Windvogel, Johan Louw, Christo J. F. Muller

**Affiliations:** ^1^ Biomedical Research and Innovation Platform, Grants, Innovation and Product Development Unit South African Medical Research Council Tygerberg South Africa; ^2^ Centre for Cardio‐metabolic Research in Africa (CARMA), Division of Medical Physiology, Faculty of Medicine and Health Sciences Stellenbosch University Tygerberg South Africa; ^3^ Division of Medical Physiology, Faculty of Health Sciences Stellenbosch University Tygerberg South Africa; ^4^ Department of Biochemistry and Microbiology University of Zululand Kwadlangezwa South Africa

**Keywords:** atorvastatin, C3A cells, hepatotoxicity, herb‐drug interaction, Rooibos

## Abstract

Rooibos (*Aspalathus linearis*) has various health benefits. Two case studies have associated chronic Rooibos consumption with conventional prescription medications, including atorvastatin (ATV), with hepatotoxicity. Statins act by inhibiting hydroxymethylglutaryl‐coenzyme A reductase, a rate‐limiting enzyme in cholesterol synthesis. Although rare, statins are potentially hepatotoxic. The aim was to investigate interactions between aspalathin‐rich Rooibos extract GRT™ and ATV‐induced hepatotoxicity in C3A liver cells cultured with and without palmitate. Effects of co‐treatment of GRT + ATV on cell viability, oxidative stress, apoptosis, mitochondrial integrity, and cellular reactive oxygen species (ROS) production were assessed. Significantly increased ROS production was observed in cells exposed to ATV and palmitate. Combination therapy of GRT + ATV also showed significant increases in ROS production. Under palmitate‐treated conditions, ATV‐induced significant apoptosis which was not ameliorated by GRT + ATV co‐treatment. Despite studies purporting hepatoprotection from Rooibos, our study showed that GRT was unable to modulate ATV‐induced hepatotoxic effects in this model.

## Graphical abstract

Effects of rooibos extract (GRT) and atorvastatin (ATV) treatments on reactive oxygen species (ROS) production in C3A cells without palmitate and cells treated with 500 µM palmitate for 24 hrs. The addition of GRT failed to ameliorate ROS production induced by ATV treatment.

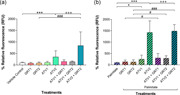

## 1 INTRODUCTION

*Aspalathus linearis*, commonly known as Rooibos, is a fynbos leguminous shrub indigenous to the Western and Northern Cape Provinces of South Africa, recognized for its health benefits. Scientific evidence confers multiple health benefits to Rooibos, such as anti‐inflammatory (Lee & Bae, 2015), antioxidant (Joubert & de Beer, 2012), hepatoprotective (Ajuwon, Oguntibeju, & Marnewick, 2014; Canda, Oguntibeju, & Marnewick, 2014; Kucharská et al., 2004; Ulicná et al., 2003), lipid‐lowering (Bursill, Abbey, & Roach, 2007; Marnewick et al., 2011, Orlando et al., 2019), antidiabetic, and hypoglycaemic (Johnson et al., 2016; Kawano et al., 2009; Mazibuko et al., 2013, 2015; Muller et al., 2012; Son, Minakawa, Miura, & Yagasaki, 2013) effects. These health benefits are attributed to the presence of various polyphenolic compounds, including aspalathin (Ajuwon, Katengua‐Thamahane, Van Rooyen, Oguntibeju, & Marnewick, 2013; McKay & Blumberg, 2009; Ulicná et al., 2003).

Given the growing use of natural health products to enhance health, the likelihood of supplementing chronic prescriptions with natural medications is increasing. However, information on the safety of combining natural products and pharmaceutical products is sparse and the potential for herb‐drug interactions is of clinical concern (World Health Organization, 2019). A review by Awortwe et al. (2018) highlighted that patients on statins and/or warfarin therapy presented with clinically significant complications after concomitant use of herbal products, including green tea. Anecdotally, Rooibos tea consumption is safe. However, two case studies exist that associate treatment of rituximab and daily administration of prednisolone with Rooibos (Sinisalo, Enkovaara, & Kivistö, 2010), or a Rooibos and Buchu tea combination taken with oral steroids and long‐term atorvastatin (ATV) use (Engels, Wang, Matoso, Maidan, & Wands, 2013), with hepatotoxicity. Although, causality could not be attributed to Rooibos use, the potential risk for herb‐drug interaction needs to be investigated

ATV is considered a “blockbuster drug” as the best‐selling prescription drug in history, with lifetime sales of USD 148,744 million between 1996 and 2016 (editorial in King, 2013; The Lancet, 2011). Statins are major chronic prescription drugs worldwide that are administered to lower increased cholesterol levels in patients with increased risk of developing cardiovascular disease. They act by competitively inhibiting hydroxymethylglutaryl‐coenzyme A (HMG‐CoA) reductase, the first and key rate‐limiting enzyme of the cholesterol biosynthetic pathway (Björnsson, 2016; Björnsson, Jacobsen, & Kalaitzakis, 2012; Pal, Ghosh, Ghosh, Bhattacharyya, & Sil, 2015; Schaefer & Asztalos, 2006). Although rare, statin usage has resulted in serious side‐effects in some patients, the most severe of which include new‐onset diabetes, myalgia and myopathy, as well as the potential of rhabdomyolysis or hepatotoxicity (Björnsson, 2016; Clarke & Mills, 2006; Pal et al., 2015). The presence of these side‐effects or even the potential thereof, often causes patients to cease statin treatment and pursue alternative treatment options. A retrospective study (between 2000 and 2008) by Zhang et al. (2013) showed that, in a cohort of 107,835 patients, 17.4% experienced statin‐related side‐effects, and 59.2% of which discontinued statin treatment as a result (Zhang et al., 2013). These patients may be self‐medicating with supplements that exert their own health and risk‐profile modulating effects, Rooibos extract supplementation, as an example. A review by Kraft (2009) showed that the prevalence of patients making use of complementary or alternative medicine ranges from 24% to 70% in various studies in the United States and Canada (Kraft, 2009). A study by Wazaify, Alawwa, Yasein, Al‐Saleh, and Afifi (2013) showed that 11.6% of the 700 participants made use of complementary and alternative medicines, 27.2% of who presented with dyslipidaemia (Wazaify et al., 2013). The Allied Health Care Professions of South Africa estimated that, in 2010, as much as 19% of the South African population made use of traditional and complementary medicine (World Health Organization, 2019). Preclinically, Rooibos is hepatoprotective against chemically‐induced hepatotoxicity and hepatic injury (i.e., through administration of carbon tetrachloride [CCl_4_^−^], lipopolysaccharide [LPS], or *tert*‐butyl hydroperoxide (*t*‐BHP); Ajuwon et al., 2014; Canda et al., 2014; Kucharská et al., 2004, respectively), but its effect on ATV‐induced hepatotoxicity is not known. It is therefore of clinical importance to understand whether these interactions could pose an added risk for hepatotoxicity, or, contrastingly, whether Rooibos is able to ameliorate ATV‐induced hepatotoxic damage.

This study aimed to induce acute hepatotoxicity using ATV in C3A liver cells under a normal and simulated hyperlipidaemic condition, to measure the extent of hepatotoxic damage in terms of various cellular parameters, and to investigate the potential hepatoprotective effects of Rooibos in this context. Exposure of C3A liver cells to high‐dose ATV was used as a model to assess whether ATV‐induced hepatotoxicity could be ameliorated by the hepatoprotective effects of an aspalathin‐rich Rooibos extract (Afriplex GRT™).

## 2 METHODS AND MATERIALS

### 2.1 Reagents

The human‐derived hepatocarcinoma cell line C3A (cat# CRL‐10741, RRID: CVCL_1098; ATCC) was purchased from the American Type Culture Collection (ATCC). Eagle's modified essential medium (Lonza, MD), 10% vol/vol fetal bovine serum (Gibco; catalog no.: 10500064; Thermo Fisher Scientific, Johannesburg, South Africa) and 1% vol/vol l‐glutamine (Lonza, MD), the combination thereof hereafter referred to as growth medium. Cells were seeded at a density of 11 × 10^4^ cells per ml and incubated in humidified air at 37°C with 5% CO_2_. Replacement of culture media was done every 2–3 days. Cells were sub‐cultured to 70–80% confluence (ATCC, n.d.). Palmitate was prepared in ethanol, and thereafter palmitate treatment was prepared in growth medium consisting of 2 mg/ml bovine serum albumin (fraction V, BSAV‐RO; Roche, Sigma‐Aldrich, Johannesburg, South Africa) and 4.7 g/L sodium bicarbonate (catalog no.: S3817; Sigma‐Aldrich, Stanheim, Germany) and left to conjugated at 37°C (Mazibuko et al., 2013, 2015).

An aspalathin‐rich unfermented Rooibos extract (Afriplex GRT™) was obtained from Afriplex (Pty) Ltd (Paarl, South Africa), ATV was obtained from Sigma‐Aldrich (catalog no.: PHR1422), and palmitate was obtained from Sigma‐Aldrich (catalog no.: P0500). These, and all other chemicals for experimental use were of cell culture grade. Stock solutions were prepared in dimethyl sulfoxide (DMSO) and final working concentrations of treatments contained not more than 0.25% vol/vol DMSO.

3‐(4, 5‐Dimethylthiazol‐2‐yl)‐2,5‐diphenyl tetrazolium bromide (MTT; catalog no.: M5655), 2′,7′‐dichlorofluorescein (DCF; catalog no.: D6883), 5,5′,6,6′‐tetrachloro‐1,1′,3,3′‐tetraethylbenzimidazolylcarbocyanine iodide (JC‐1; catalog no.: T3168), and propidium iodide (PI; catalog no.: P4170) were obtained from Sigma, Stanheim, Germany.

Annexin V (catalog no.: A13199) and caspase 3/7 (catalog no.: C10423) were obtained from Thermo Fisher Scientific, Johannesburg, South Africa.

### 2.2 Culturing of C3A cells

**Figure 1:**
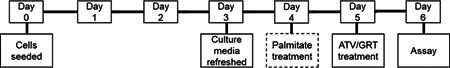
Schematic representation of treatment timeline for assays

C3A liver cells, with palmitate or without palmitate, were exposed to ATV (10 and 25 µM) and Afriplex GRT™ (0.01 and 0.1 mg/ml) and a combination thereof (10 µM ATV + 0.01 mg/ml GRT, 10 µM ATV + 0.1 mg/ml GRT, or 25 µM ATV + 0.1 mg/ml GRT) for 24 hr. Pretreatment with palmitate (500 µM) for 24 hr was used to simulate hyperlipidaemia in vitro (Figure 1).

### 2.3 MTT assay

Cellular metabolic activity was assessed using an MTT cell viability assay (Mosmann, 1983). C3A cells were seeded at a density of 11 × 10^4^ cells per ml in 200 µl growth medium per well in a 96‐well plate and cultured and treated as per Figure 1. For the assay, cells were washed with pre‐warmed phosphate‐buffered saline before being incubated with 50 µl 2 mg/ml MTT for 30 min at 37°C. Following incubation, the MTT was aspirated and 200 µl DMSO and 25 µl Sorenson's Buffer (pH 7.4) was added. Spectrophotometric measurements were recorded at OD_570_ using a BioTek ELx800 absorbance microplate reader. Results were generated using Gen5 (RRID:SCR_017317) version 1.05. Three replicates per treatment were assessed in three independent experiments.

### 2.4 DCF assay

Oxidative stress was assessed by DCF staining (Kalyanaraman et al., 2012). C3A cells were seeded at a density of 11 × 10^4^ cells per ml in 200 µl growth medium per well in a black 96‐well clear bottom plate and cultured and treated as per Figure 1. ROS production was determined using an endpoint fluorogenic DCF assay incubated for 30 min (37°C in 5% CO_2_ in humified air). Fluorescent readings were recorded using a SpectraMax i3x multi‐mode microplate reader at an excitation wavelength of 485 nm and an emission wavelength of 535 nm. Results were generated on SoftMax Pro Data Acquisition and Analysis Software (RRID:SCR_014240), version 7.0.2. Three replicates per treatment were assessed in three independent experiments. Reactive oxygen species production was calculated relative to cell viability which was assessed by an MTT assay.

### 2.5 JC‐1 assay

Mitochondrial membrane integrity was assessed by utilizing the JC‐1 stain (Di Lisa et al., 1995; Reers, Smith, & Chen, 1991). C3A cells were seeded at a density of 11 × 10^4^ cells per ml in 1 ml growth medium per well in a 24‐well plate and cultured and treated as per Figure 1. Post treatment, treatment media was aspirated, replaced with 500 µl of 2 µM JC‐1 stain, and incubated at 37°C for 30 min. Mitochondrial membrane potential was assessed with a BD Accuri™ C6 flow cytometer (RRID:SCR_014422). Two replicates per treatment were assessed in three independent experiments.

### 2.6 Annexin V/PI assessment

Apoptosis was assessed with annexin V/PI staining (Van Engeland, Ramaekers, Schutte, & Reutelingsperger, 1996; Vermes, Haanen, Steffens‐Nakken, & Reutelingsperger, 1995). C3A cells were seeded at a density of 11 × 10^4^ cells per ml in 1 ml growth medium per well in a 24‐well plate and cultured and treated as per Figure 1. Apoptosis status was assessed with dual staining of annexin V and PI, incubated at 37°C for 30 min. Flow cytometry was performed using a BD Accuri™ C6. Two replicates per treatment were assessed in three independent experiments.

### 2.7 Caspase 3/7 activation

Apoptosis was confirmed by caspase 3/7 staining (Walsh et al., 2008). C3A cells were seeded at a density of 11 × 10^4^ cells per ml in 200 µl growth medium per well in a black 96‐well clear bottom plate and cultured and treated as per Figure 1. Apoptotic cells were stained with 10 µM caspase 3/7 reagent as per the manufacturer's instruction, incubated at 37°C for 1 hr. Fluorescent readings were recorded on a SpectraMax i3x multi‐mode microplate reader at an excitation wavelength of 502 nm and an emission wavelength of 530 nm every 15 min for 60 min. Results were generated on SoftMax Pro 7 software, version 7.2. Three replicates per treatment were assessed in three independent experiments.

### 2.8 Statistical analysis

Statistical analysis was performed using GraphPad Prism 8.2.1 (RRID:SCR_002798) and data were compared using one‐way analysis of variance, followed by a Tukey post hoc test. Data are presented as mean ± standard deviation and *p* values of ≤.05 were considered statistically significant.

## 3 RESULTS

### 3.1 Mitochondrial dehydrogenase activity

**Figure 2:**
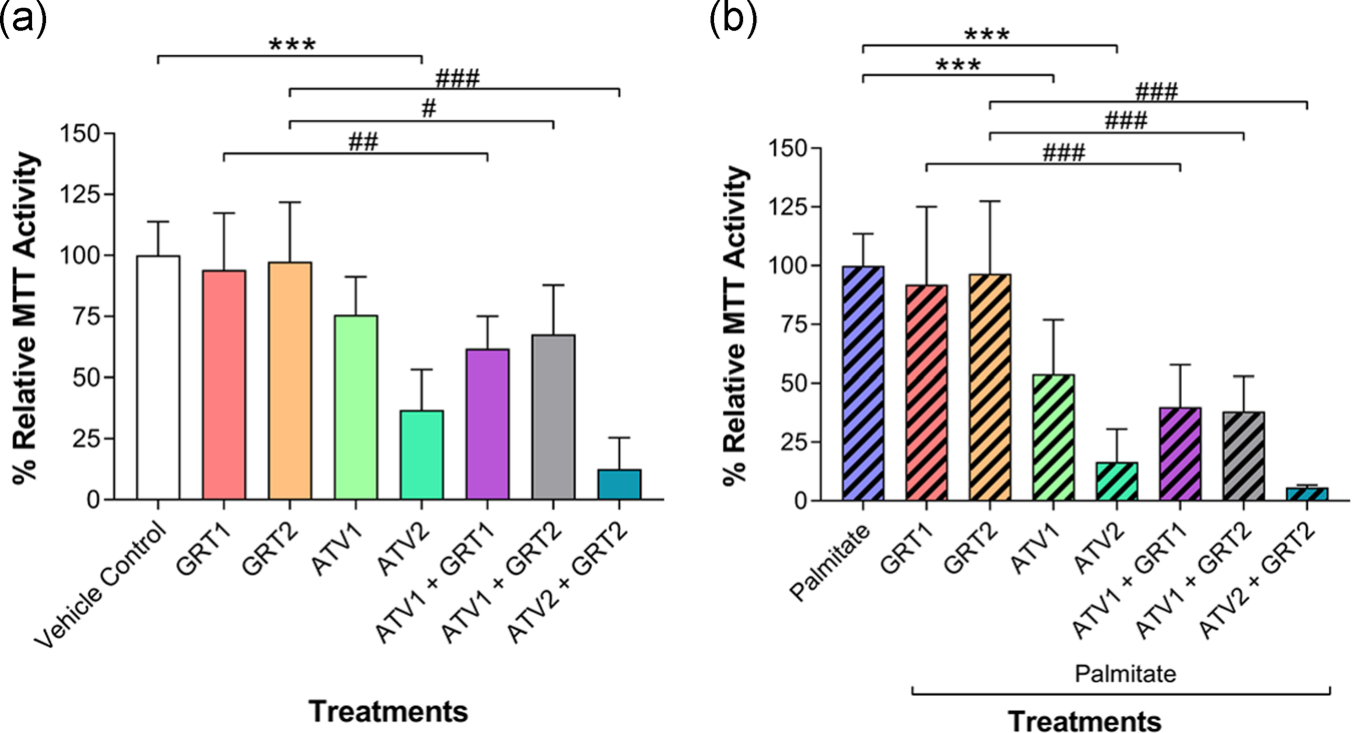
(a) Effects of GRT and ATV treatments on MTT mitochondrial dehydrogenase activity in C3A cells without palmitate and (b) cells treated with 500 µM palmitate for 24 hrs cells. Data were expressed as mean ±* SD* (*n* = 3). **p < .01, ***p < .001 versus vehicle control or palmitate; ^#^ *p *< .05, ^##^ *p *< .01, ^###^ *p *< .001 versus respective GRT monotherapy. GRT1 = 0.01 mg/ml and GRT2 = 0.1 mg/ml aspalathin‐rich unfermented Rooibos extract (Afriplex GRT™); ATV1 = 10 µM and ATV2 = 25 µM atorvastatin. ATV, atorvastatin; MTT, 3‐(4, 5‐dimethylthiazol‐2‐yl)‐2,5‐diphenyl tetrazolium bromide; *SD*, standard deviation

Figure 2a shows that GRT treatment at the concentrations tested (GRT1 = 0.01 mg/ml and GRT2 = 0.1 mg/ml) did not affect cell viability in C3A cells not exposed to palmitate. In contrast, ATV reduced C3A cell viability in a concentration‐dependent manner. A significant decline in C3A cell viability was demonstrated after 24‐hr treatment with 25 µM ATV compared with vehicle control (36.77 ± 16.45% vs. 100.00 ± 13.84%, respectively; *p *< .001). Combination treatment of ATV (10 and 25 µM) with GRT (0.01 and 0.1 mg/ml) did not ameliorate ATV‐induced cytotoxicity. A significant difference was noted between the monotherapy treatment groups and the combination treatment groups: GRT1 (94.05 ± 23.21%) showed significantly increased (*p *< .01) MTT activity compared with ATV1 + GRT1 (61.85 ± 13.18%), whilst GRT2 (97.48 ± 24.32%) showed significantly greater activity compared with ATV1 + GRT2 (67.65 ± 20.13%; *p *< .05) and ATV2 + GRT2 (12.52 ± 12.95%; *p *< .001).

In palmitate‐treated C3A cells (experimental hyperlipidaemic condition; Figure 2b), GRT treatment did not affect cell viability. However, as was seen without palmitate, ATV‐induced significant cytotoxicity in palmitate‐treated C3A cells in a concentration‐dependent manner after 24 hr (Palmitate; 100.00 ± 13.58%; 10 µM ATV = 53.78 ± 23.20%; *p *< .001; 25 µM ATV = 16.58 ± 13.99%; *p *< .001). The adverse effect of ATV was not improved by the combination treatment with GRT at either concentration. GRT1 (91.92 ± 33.19%) increased (*p *< .001) MTT activity compared with ATV1 + GRT1 (39.92 ± 17.87%), and GRT2 (96.56 ± 30.92%) showed higher activity compared with ATV1 + GRT2 (38.05 ± 14.91%; *p *< .001) and ATV2 + GRT2 (5.65 ± 1.03%; *p *< .001).

### 3.2 Oxidative stress

**Figure 3:**
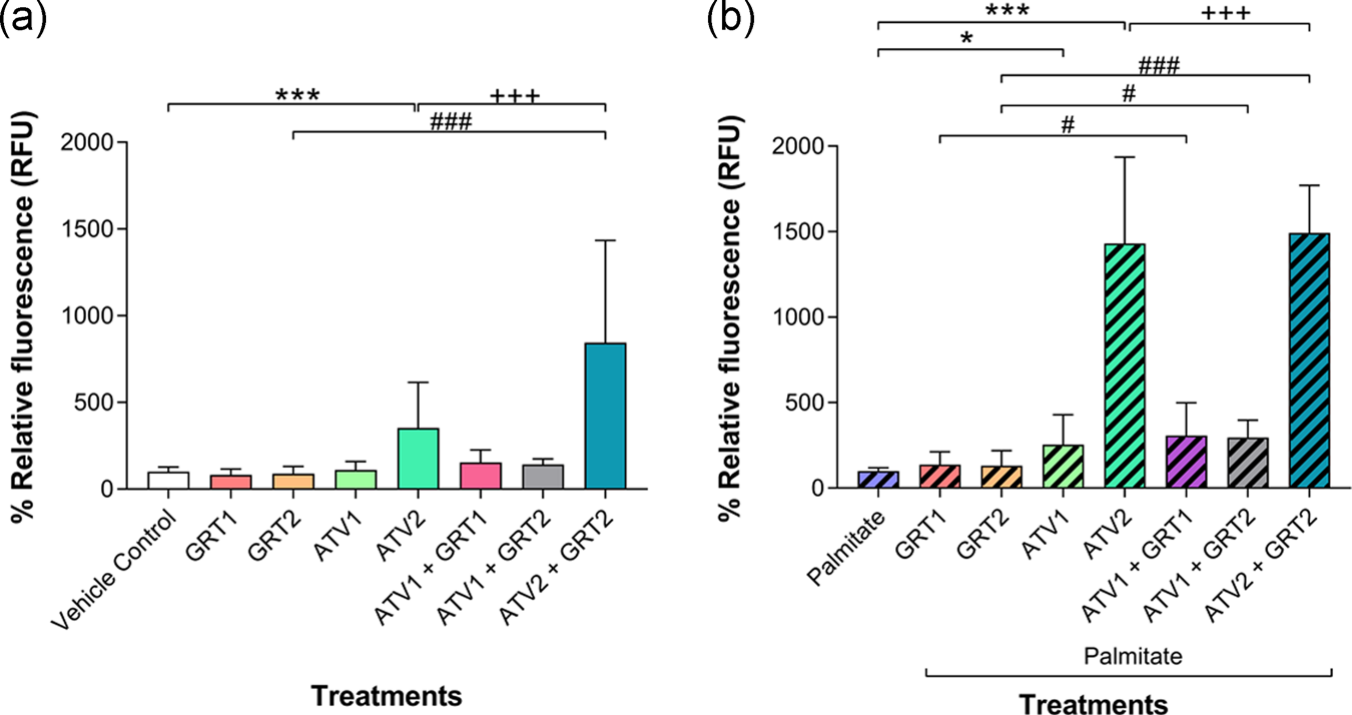
(a) Effects of GRT and ATV treatments on ROS production in C3A cells without palmitate and (b) cells treated with 500 µM palmitate for 24 hrs cells. Data were expressed as mean ± *SD* (*n* = 3). *p < .05, **p < .01, ***p < .001 versus vehicle control or palmitate; ^#^ *p *< .05, ^###^ *p *< .001 versus respective GRT monotherapy; ^+++^ *p *< .001 versus respective ATV monotherapy. GRT1 = 0.01 mg/ml and GRT2 = 0.1 mg/ml aspalathin‐rich unfermented Rooibos extract (Afriplex GRT™); ATV1 = 10 µM and ATV2 = 25 µM atorvastatin. ATV, atorvastatin; ROS, reactive oxygen species; *SD*, standard deviation

In cells not exposed to palmitate as shown in Figure 3a, GRT had no effect on intracellular ROS production, whilst ATV2 (353.10 ± 262.70%) significantly increased (*p *< .001) intracellular ROS compared with the vehicle control (100.00 ± 27.52%). Co‐treatment of GRT + ATV had a significant (*p *< .001) additive effect to the ATV‐induced intracellular ROS production compared with GRT2 (89.96 ± 40.96%) and ATV2 monotherapy (ATV2 + GRT2 = 845.00 ± 589.60%).

With palmitate treatment (Figure 3b), ATV‐induced a concentration‐dependent increase of intracellular ROS, with 10 µM ATV (254.90 ± 175.10%; *p *< .05) and 25 µM ATV (1431.00 ± 504.20%; *p *< .001) significantly increasing intracellular ROS compared with the palmitate control (100.00 ± 19.07%). In combination, GRT at the concentrations used did not moderate ATV‐induced ROS production. A significant difference was seen between GRT monotherapy (137.80 ± 74.29% for GRT1 and 131.40 ± 87.18% for GRT2) compared with their respective combination treatment groups: ATV1 + GRT1 = 306.80 ± 192.20% (*p *< .05); ATV1 + GRT2 = 296.10 ± 102.00% (*p *< .05); ATV2 + GRT2 = 1493.00 ± 278.40% (*p *< .001). However, there was a significant difference (*p *< .001) between the ATV2 and ATV2 + GRT2 treatment groups.

### 3.3 Mitochondrial membrane potential assessment

**Figure 4:**
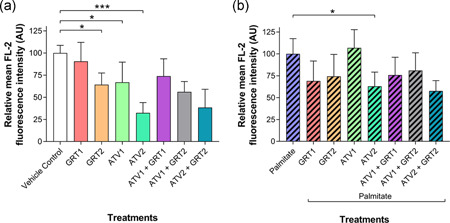
(a) Effects of GRT and ATV treatments on mitochondrial membrane potential in C3A cells without palmitate and (b) cells treated with 500 µM palmitate for 24 hrs. Data were expressed as mean ± *SD* (*n* = 3). *p < .05, ***p < .001 versus vehicle control or palmitate. GRT1 = 0.01 mg/ml and GRT2 = 0.1 mg/ml aspalathin‐rich unfermented Rooibos extract (Afriplex GRT™); ATV1 = 10 µM and ATV2 = 25 µM atorvastatin. ATV, atorvastatin; *SD*, standard deviation

In cells not exposed to palmitate, Figure 4a, GRT2 (64.28 ± 13.16%; *p *< .05), ATV1 (66.99 ± 22.73%; *p *< .05), and ATV2 (32.48 ± 11.55%; *p *< .001) treatment significantly reduced relative mean fluorescence intensity compared with the vehicle control (100.00 ± 8.76%). Figure 4b shows that, in palmitate‐treated cells, ATV2 had a significant effect on mitochondrial membrane potential compared with the palmitate control (63.02 ± 16.17% vs. 100.00 ± 17.52%, respectively; *p *< .05).

### 3.4 Apoptosis assessment

**Figure 5:**
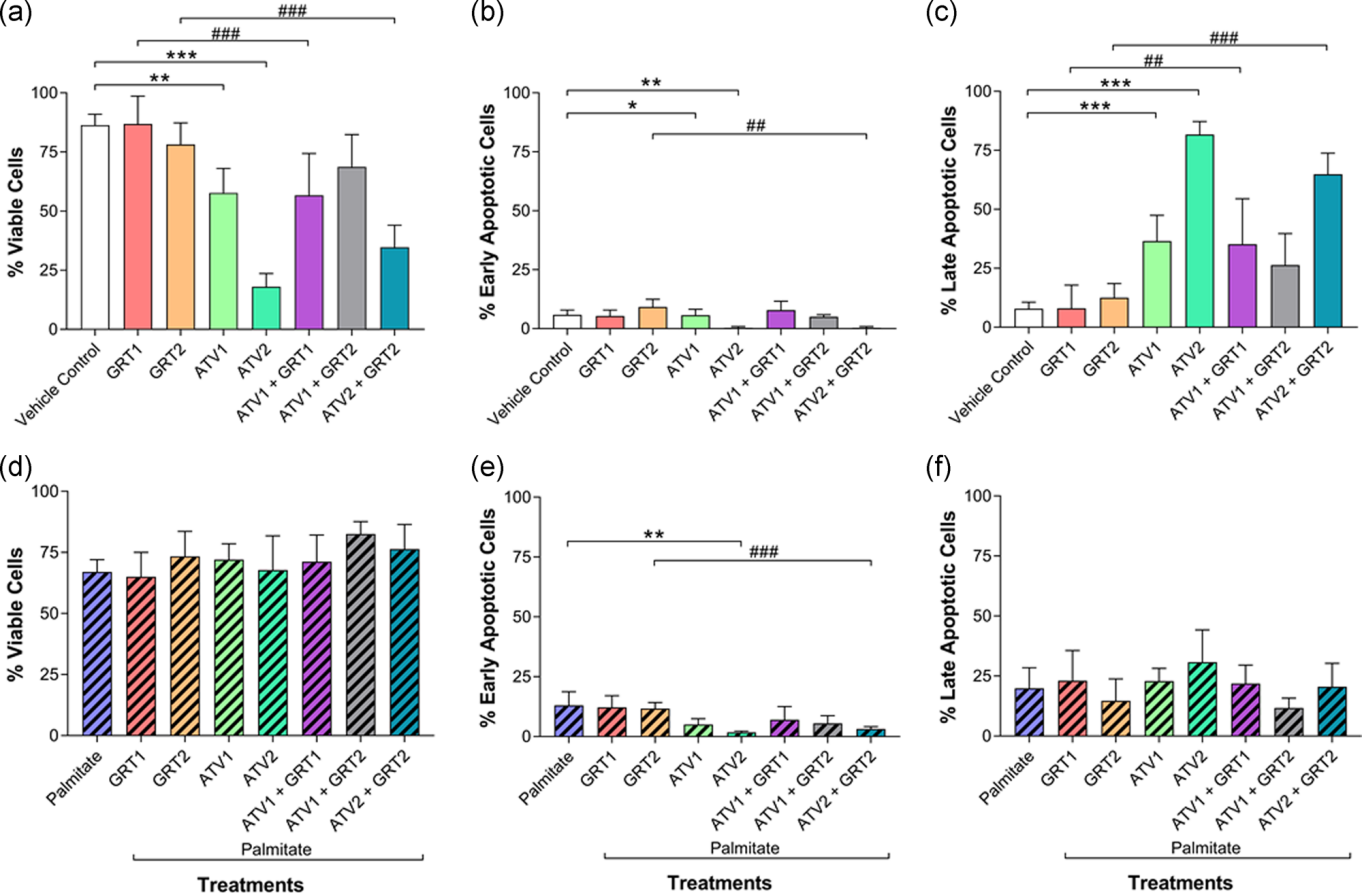
(a–c) Effects of GRT and ATV treatments on apoptosis status in C3A cells without palmitate and (d–f) cells treated with 500 µM palmitate for 24 hrs. Percentage of cell population in each viability phase was determined and data were expressed as mean ± *SD* (*n* = 3). *p < .05, **p < .01, ***p < .001 versus vehicle control or palmitate; ^##^ *p *< .01, ^###^ *p *< .001 versus respective GRT monotherapy. GRT1 = 0.01 mg/ml and GRT2 = 0.1 mg/ml aspalathin‐rich unfermented Rooibos extract (Afriplex GRT™); ATV1 = 10 µM and ATV2 = 25 µM atorvastatin. ATV, atorvastatin; *SD*, standard deviation

In the absence of palmitate (Figure 5a), the addition of ATV1 (57.67 ± 10.41%; *p *< .01) and ATV2 (18.00 ± 5.62%; *p *< .001) significantly decreased the number of viable cells compared with the vehicle control (86.33 ± 4.63%). The addition of GRT at either concentration was unable to protect these cells against ATV toxicity. GRT1 monotherapy significantly increased the percentage of viable cells compared with ATV1 + GRT1 combination therapy (86.83 ± 11.81% vs. 56.67 ± 17.68%, respectively). Similarly, GRT2 monotherapy significantly increased (*p *< .001) the percentage of viable cells compared with ATV2 + GRT2 combination therapy (78.17 ± 9.09% vs. 34.67 ± 9.40%, respectively).

There was a significant difference (*p *< .01) in the percentage of early apoptotic cells when GRT2 (9.17 ± 3.25%) and ATV2 + GRT2 (0.33 ± 0.52%) were compared (Figure 5b). The ATV1 group (5.67 ± 2.50%; *p *< .05) and ATV2 group (0.33 ± 0.52%; *p *< .01) showed significantly less prevalence of early apoptosis as compared with the vehicle control group (5.83 ± 2.04%). The combination of GRT1 and GRT2 did not significantly improve ATV1 or ATV2‐induced apoptosis.

Cells in late apoptosis, not treated with palmitate (Figure 5c), showed inverse results to the viable cells, not treated with palmitate (Figure 5a), and showed similar statistical comparisons between treatment groups. The ATV1 group (36.50 ± 10.93%) showed significantly (*p *< .001) more late apoptotic cells compared the vehicle control (7.83 ± 2.79%), as did the ATV2 group (81.67 ± 5.57%; *p *< .001), which showed the greatest percentage of late apoptotic of all the treatment groups in the normal condition. Compared with ATV1 + GRT1 (35.17 ± 19.28%), GRT1 (8.00 ± 9.90%) showed a significant (*p *< .01) decrease in the prevalence of late apoptotic cells. The GRT2 group (12.50 ± 6.06%) showed a significant (*p *< .001) decrease compared with ATV2 + GRT2 (64.83 ± 8.98%).

In terms of the percentage of viable cells in the simulated hyperlipidaemic condition, there was no statistical differences between any of the different treatment groups (Figure 5d). Although insignificant, GRT1 showed the lowest percentage of viable cells (65.00 ± 10.04%) while ATV1 + GRT2 showed the greatest percentage of viable cells (82.50 ± 5.09%). In terms of early apoptosis in the hyperlipidaemic condition (Figure 5e), ATV2 (1.75 ± 0.50%) showed a significantly (*p* < .01) decreased percentage of cells relative to the palmitate control group (13.00 ± 5.73%). Further, ATV2 + GRT2 (3.17 ± 0.98%) showed significantly decreased (*p *< .001) percentage of early apoptotic cells compared with the GRT2 monotherapy group (11.67 ± 2.58%). Figure 5f showed no significant differences between any of the treatment groups.

### 3.5 Caspase activation

**Figure 6:**
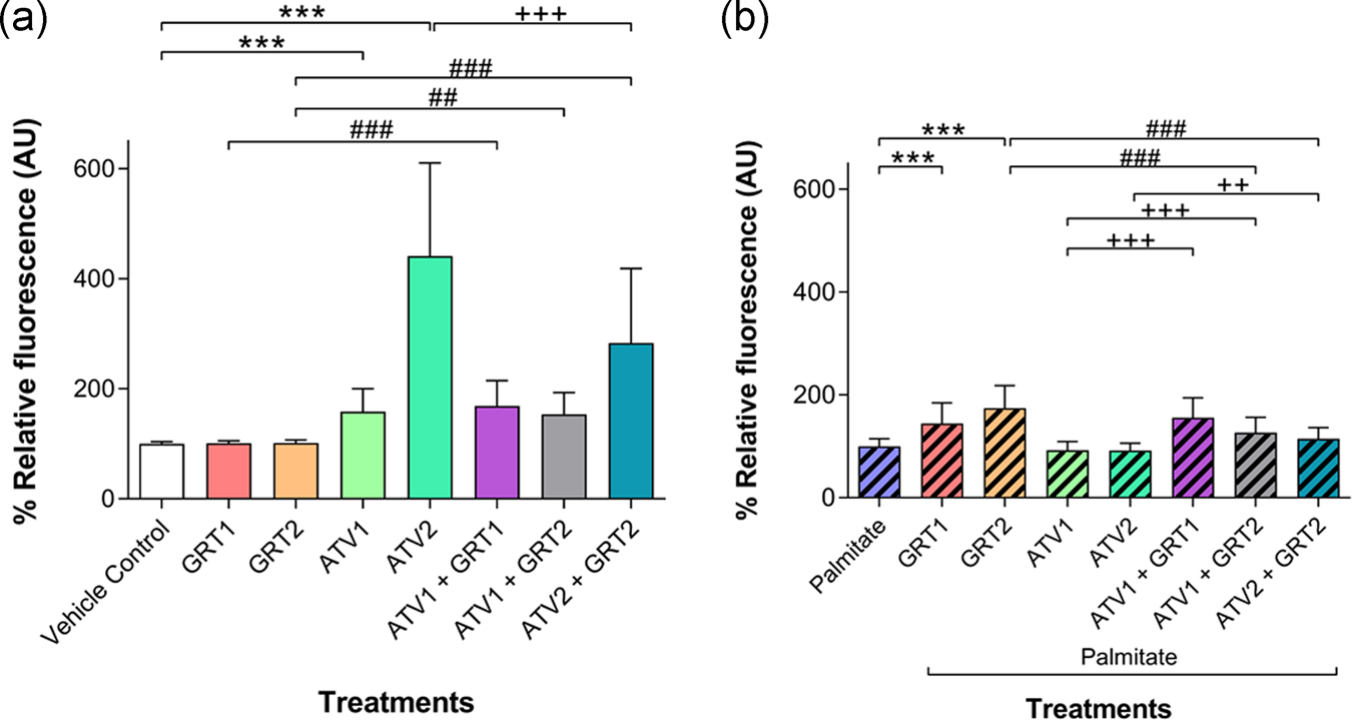
(a) Effects of GRT and ATV treatments on caspase activation in C3A cells without palmitate and (b) cells treated with 500 µM palmitate for 24 hrs. Data were expressed in mean ±* SD* (*n* = 3). **p < .01, ***p < .001 versus vehicle control or palmitate; ^##^ *p *< .01, ^###^ *p *< .001 versus respective GRT monotherapy; ^++^ *p *< .01, ^+++^ *p *< .01 versus respective ATV monotherapy. GRT1 = 0.01 mg/ml and GRT2 = 0.1 mg/ml aspalathin‐rich unfermented Rooibos extract (Afriplex GRT™); ATV1 = 10 µM and ATV2 = 25 µM atorvastatin. ATV, atorvastatin; *SD*, standard deviation

Figure 6a shows that, in the normal condition, the ATV1 (158.50 ± 41.78%) group alone showed significantly increased (*p *< .001) caspase 3/7 activity compared with the vehicle control (100.00 ± 3.67%), as did the ATV2 group (441.00 ± 169.20%; *p *< .001). The difference between GRT1 (100.90 ± 4.44%) and ATV1 + GRT1 (168.70 ± 45.81%) was significant (*p < .001*), as was the difference between GRT2 (101.80 ± 41.78%) and ATV1 + GRT2 (153.50 ± 39.40%; *p *< .01) and GRT2 and ATV2 + GRT2 (283.20 ± 135.00%). There was also a significant (*p *< .001) difference between ATV2 and ATV2 + GRT2.

Figure 6b shows that the GRT1 (144.20 ± 40.45%) and GRT2 (174.20 ± 43.65%) groups showed significantly more (*p *< .001) caspase 3/7 activity compared with the palmitate control group (100.00 ± 14.83%) in the hyperlipidaemic condition. The addition of GRT2 had a seemingly additive effect on the ATV monotherapy groups (92.24 ± 16.85% and 91.85 ± 13.82% for ATV1 and ATV2, respectively) as compared with their combination therapy counterparts: ATV1 + GRT1 = 155.50 ± 38.90% (*p *< .001); ATV1 + GRT2 = 126.20 ± 29.90% (*p *< .001); and ATV2 + GRT2 = 114.70 ± 21.82% (*p *< .01). Similarly, GRT2 monotherapy showed significantly increased (*p *< .001) caspase 3/7 activity compared with the ATV1 + GRT2 and ATV2 + GRT2 combination therapy groups.

## 4 DISCUSSION

ATV is pharmacologically classified as a high‐intensity statin, and was chosen as it appears to be the predominant statin associated with hepatotoxicity in patients (Björnsson, 2016). Rooibos (*Aspalathus linearis*) is commonly prepared as a herbal infusion and has a number of health properties including antioxidant, hepatoprotective, and metabolic effects (Ajuwon et al., 2013; Mazibuko‐Mbeje et al., 2019; Waisundara & Hoon, 2015). Given the prevalent use of herbal preparations as complementary and even alternative treatments to modern medicine, we explored the use of GRT, an aspalathin‐rich extract of Rooibos, alone and in combination with ATV in an in vitro model of palmitate‐induced dyslipidaemia. The high‐performance liquid chromatography chemical characterization of the aspalathin‐rich unfermented Rooibos extract GRT™ revealed an aspalathin content of 12% (Patel et al., 2016) which is much higher than expected from the daily consumption of fermented or unfermented Rooibos tea. Joubert and de Beer (2012) considered the phenolic content of Rooibos at a “cup of tea” concentration as well as at the concentrations of an industrial extract, which is accepted to be the equivalent of six cups of Rooibos tea. The study showed that the average aspalathin content of a “cup of tea” is approximately 0.53%, and typically 7% in industrial extracts (Joubert & de Beer, 2012). Furthermore, ATV is lipophilic and passively diffuses across the cell membrane. The addition of palmitate pretreatment to the study design to simulate dyslipidaemia in vitro is representative of the clinical condition wherein statin therapy is likely to be prescribed. The presence of these metabolic alterations leaves the liver cells more susceptible to further injury by ATV than normal liver cells (Koh, Sakuma, & Quon, 2011). The palmitate concentrations selected (500 µM) was within a literature‐relevant range (Abu Bakar & Tan, 2017; Mazibuko et al., 2015; Zezina et al., 2018).

Mitochondrial membrane potential was assessed using a JC‐1 assay, which was confirmed with a DCF assay assessing ROS generation. Positive results in these assays were confirmed in terms of apoptosis activation, with specific consideration on membrane integrity (annexin V/PI staining) and caspase activation.

In cells not pretreated with palmitate, both 10 and 25 µM ATV caused a significant decrease in relative MTT activity, while GRT alone showed no difference at the concentrations used after 24 hr. In combination, GRT did not modulate the toxicity induced by ATV in terms of MTT activity. The same trend was noted in the palmitate‐treated cells. Taken together, this suggests that GRT did not have a modulating effect on ATV‐induced toxicity in terms of MTT activity.

In the normal condition, as expected GRT did not induce ROS production. The higher concentration of ATV (25 µM) increased ROS production, and co‐treatment with 0.1 mg/ml GRT further exacerbated this effect. In the hyperlipidaemic condition, a similar trend was seen: GRT alone did not induce ROS production, however 25 µM ATV treatment, both alone and in combination with GRT, increased ROS. Shu et al. (2016) attributed ATV‐induced hepatotoxicity in the hyperlipidaemic condition to increased ROS production. The antioxidant potential of Rooibos has been well documented and has been shown to increase the activity of endogenous antioxidant systems, as well as having ROS scavenging capabilities (Ajuwon et al., 2013, 2014; Kucharská et al., 2004; Marnewick, Joubert, Swart, van der Westhuizen, & Gelderblom, 2003; Ulicná et al., 2003). In this study, GRT was unable to ameliorate the ATV‐induced ROS, suggesting that GRT was unable to protect the cells against ATV‐induced ROS.

In contrast, other studies have demonstrated hepatoprotective effects for Rooibos against CCl_4_^−^ (Kucharská et al., 2004), LPS (Ajuwon et al., 2014), and *t*‐BHP (Canda et al., 2014) in vivo. It can be speculated that whilst our results conflicted with those of the other studies, this could be explained by fundamental differences in experimental design. Although the toxic mechanisms differ, CCl_4_^−^, LPS, and *t*‐BHP induce excessive oxidative stress that overcome the endogenous cellular antioxidant capacity, causing lipid peroxidation, increased mitochondrial permeability, endoplasmic reticulum stress, and cellular membrane dysfunction. The hepatotoxic effect of ATV is potentially caused by oxidative stress and Ca^2+^‐induced mitochondrial membrane permeability transition, inhibition of HMG‐CoA reductase by statins affects many other vital cellular processes (Maciejak et al., 2013). Importantly, by inhibiting mevalonate pathway, apart from cholesterol synthesis, statins also suppress nonsterol isoprenoid pathways, involving ubiquinone biosynthesis (CoQ_9/10_). This is an important redox component of the mitochondrial electron transport chain, responsible for synthesizing ATP. Reducing the biosynthesis of CoQ_9/10_ in the liver affects mitochondrial oxygen consumption and increases cellular ROS production, thought to be a major causal factor for statin‐induced adverse effects (Rundek, Naini, Sacco, Coates, & DiMauro, 2004).

Along with increased ROS, high‐dose ATV decreased mitochondrial membrane potential in both the normal and palmitate‐treated C3A cells. GRT could not attenuate the toxicity of the ATV in terms of mitochondrial membrane potential changes. The loss of mitochondrial membrane integrity and subsequent release cytochrome C from the inner membrane of the mitochondrion initiates the intrinsic apoptotic pathway culminating in the activation of caspase 3/7 (Brentnall, Rodriguez‐Menocal, De Guevara, Cepero, & Boise, 2013). As expected, caspase 3/7 activity was significantly increased by ATV, particularly at 25 µM. These results are in agreement with Docrat, Nagiah, Krishnan, Naidoo, and Chuturgoon (2018), who, using a similar assay to assess caspase activity, demonstrated a significant increase in ATV‐induced caspase activation in HepG2 cells, of which C3A cells are a sub‐clone, treated with 1.2 mM ATV. In vivo, Pal et al. (2015) demonstrated increased caspase activation in healthy rats treated with ATV at concentrations of 10 mg/kg/day. The combination of ATV2 + GRT2 showed significantly less caspase 3/7 activity compared with the ATV2 group alone, suggesting that GRT had a modulating effect on ATV‐induced caspase activity under normal conditions. However, a protective effect of GRT could not be confirmed by flow cytometry using annexin V/PI staining for apoptosis.

## 5 CONCLUSION

This study found that ATV was concentration‐ and time‐dependently hepatotoxic to C3A liver cells and this hepatotoxic effect was exacerbated by the addition of palmitate pretreatment. ATV toxicity was associated with decreased mitochondrial membrane potential, increased ROS production, as well as increased caspase 3/7 activity. GRT was unable to protect against the ATV‐induced mitochondrial dysfunction and the consequent induced toxicity. The findings from the current study suggest that concurrent supplementation with GRT appears to be ineffective at protecting C3A liver cells against ATV‐induced toxicity in vitro. However, given the complexity of bioavailability and pharmacokinetics of complex mixtures, such as plant extracts, it is difficult to accurately extrapolate in vitro results to a clinical setting. Despite this, our findings suggest that it is unlikely that GRT will exhibit sufficient hepatoprotective effects in patients with ATV‐related hepatotoxicity.

## CONFLICT OF INTERESTS

The authors hereby confirm that Professors Louw and Muller have a research interest in Afriplex GRT, a pharmaceutical grade GMP aspalathin‐enriched unfermented rooibos extract.

## Data Availability

The data that support the findings of this study are available from the corresponding author upon reasonable request.
